# Spontaneous Ejaculation in a Wild Indo-Pacific Bottlenose Dolphin (*Tursiops aduncus*)

**DOI:** 10.1371/journal.pone.0072879

**Published:** 2013-08-28

**Authors:** Tadamichi Morisaka, Mai Sakai, Kazunobu Kogi, Akane Nakasuji, Kasumi Sakakibara, Yuria Kasanuki, Motoi Yoshioka

**Affiliations:** 1 Wildlife Research Center of Kyoto University, Kyoto, Japan; 2 Japan Society for the Promotion of Science, Tokyo, Japan; 3 Mikura Island Tourist Information Center, Tokyo, Japan; 4 Graduate School of Agriculture, Tokyo University of Agriculture and Technology, Tokyo, Japan; 5 Graduate School of Bioresources, Mie University, Mie, Japan; Hokkaido University, Japan

## Abstract

Spontaneous ejaculation, which is defined as the release of seminal fluids without apparent sexual stimulation, has been documented in boreoeutherian mammals. Here we report spontaneous ejaculation in a wild Indo-Pacific bottlenose dolphin (*Tursiops aduncus*), and present a video of this rare behavior. This is the first report of spontaneous ejaculation by an aquatic mammal, and the first video of this behavior in animals to be published in a scientific journal.

## Introduction

Spontaneous ejaculation, which is defined as the release of seminal fluids in the absence of apparent sexual stimulation, has been reported in several male land mammals, including Rodentia (rats [1], hamsters [2], guinea pigs [3], mice [4]), Cetartiodactyla (mountain sheep, warthogs (reviewed in [5]), tsessebes [6]), Carnivora (domestic cats [7], spotted hyenas (reviewed in [5])), Perissodactyla (horses [8]), and Primates (chimpanzees [9], (reviewed in [10]), humans (reviewed in [11])). Spontaneous ejaculation could possibly be widespread in various animals, including humans, but has passed unrecognized because it is an unpredictable and rare behavior that lasts only a few seconds, making it difficult to observe.

The function of spontaneous ejaculation is unknown. Three (not mutually exclusive) possible “functions” of animal spontaneous ejaculation have been discussed in previous publications: (1) a type of “masturbation” as a sexual outlet and/or for the removal of surplus (or abnormal) spermatozoa [5], [10], [12]–[16]; (2) an element of sexual display [6]; (3) no clear function, or misuse of inhibitory neural control system during drowsiness and sleep [8], [11], [17]–[20].

Here, we report spontaneous ejaculation in a wild Indo-Pacific bottlenose dolphin (*Tursiops aduncus*), and present an accompanying video. This is the first report of spontaneous ejaculation in an aquatic mammal, and the first video of spontaneous ejaculation in animals to be published in a scientific journal.

The Indo-Pacific bottlenose dolphin is a small odontocete that reaches 2.7 m in length and 230 kg in weight, and appears in coastal waters from around South Africa, through the Indian Ocean, to southeast Asia and Australia [21]. Sexual maturity in male Indo-Pacific bottlenose dolphins occurs at approximately 7–8 years (reviewed in [22]). Around Mikura Island, the breeding season was estimated to be between April and October with a peak in July and August [23]. The dolphins live in fission-fusion societies, which are characterized by sex-segregation and frequent changes in group membership (reviewed in [24]). Male bottlenose dolphins engage in much higher rates of socio-sexual behavior than wild, “hypersexual” bonobos [25]. Despite many observations of socio-sexual behavior in both sexes in this species, a successful copulation or intromission has never been observed in Shark Bay, the oldest study site for this species, which was implemented in 1988 [25]. Ejaculation and actual copulation are difficult to observe even in captive dolphins [26], [27] (but see [28]). Masturbation, such as rubbing genitals on tank objects or the floor, is frequently observed in several odontocetes, including the bottlenose dolphin, spinner dolphin, killer whale, baiji, and boto [26]–[31], but never accompanied by ejaculation. At Mikura Island, we had also frequently observed masturbation and socio-sexual behaviors, but not associated ejaculation (Morisaka, Sakai, Kogi, personal communications).

## Materials and Methods

### Study sites and subjects

The video was taken underwater at about 10 m depth off Mikura Island, Japan on July 2, 2012. Indo-Pacific bottlenose dolphins around Mikura Island have almost all been identified using natural marks on the body by underwater video-identification research since 1994 (see detail in [23], [32]). Four researchers and a few other sightseers were involved in a dolphin-swimming program observed dolphins underwater, and one researcher observed dolphin behavior on the boat at the time. It was a cloudy day without rain, and the water temperature was approximately 25°C.

### Data recording

Video recording was made with an HDR-XR550V (Sony, Japan) with an attached wide conversion lens in an underwater housing system (NTF Corp, Japan). Video codec was AVCHD of 1920 pixels in width and 1080 pixels in height with 30 frames/s. When we spotted dolphins from the boat (about 7 m length), we slowly approached the group with the boat and placed the video system underwater. We did not use scuba diving equipment, but only fins, snorkels, and masks. We did not aggressively follow dolphins and finished our observations when they went away. We recorded the time, estimated number of dolphins in the group, and group behavioral state, and noted when we observe dolphins both from the boat and underwater. We classified group states into 5 categories: traveling, socializing, resting, feeding, and milling (modified [33]). Traveling was defined as dolphins swimming in the same direction near the surface at a relatively fast speed. Socializing was defined as involving sociosexual activity (e.g., mounting), chasing, playing, and/or contact behavior (e.g. flipper rubbing, contact swimming) without any consistent swim direction. Resting was defined as dolphins swimming slowly and quietly in the same direction with few breaths, usually just above the bottom in “carpet formation [34]”, where dolphins swim horizontally parallel. Feeding was defined as involving feeding activity. Milling was defined as the other group state, where dolphins were not swimming in a formation or in a consistent direction, and without any obvious behavior such as contact, feeding, etc.

### Ethics statement

This fieldwork did not involve capture or handling of animals, therefore did not require approval of animal care and use procedures. The study did not involve endangered or protected species. This study was carried out in accordance with the recommendations of the Guidelines to Study Wild Animals of the Wildlife Research Center of Kyoto University and with the voluntary regulatory rule for sustainable dolphin-swimming program made by the dolphin-swimming program operator association in Mikura Island. Mikura Island belongs to Fuji-Hakone-Izu National Park. Permission for entering protected sea area in Mikura Island was given by Mikurajima village under permit #01093. This was a collaborative work with Mikura Island Tourist Information Center.

## Results

The spontaneous ejaculation was filmed at 08:58:46 on July 2, 2012, during our underwater observation from 08:56 to 09:03 (7 min). Six minutes and 40 seconds of video were recorded during the 7-min underwater observation. The ejaculation occurred at about 10 m water depth within 200 m of Mikura Island (33°54′023″ N, 139°36′489″E), Tokyo, Japan. A 16-year-old adult male (ID number #266; Iruka-chan) was the dolphin who showed spontaneous ejaculation.

### Details of the behavior before/during the spontaneous ejaculation

**Figure 1:**
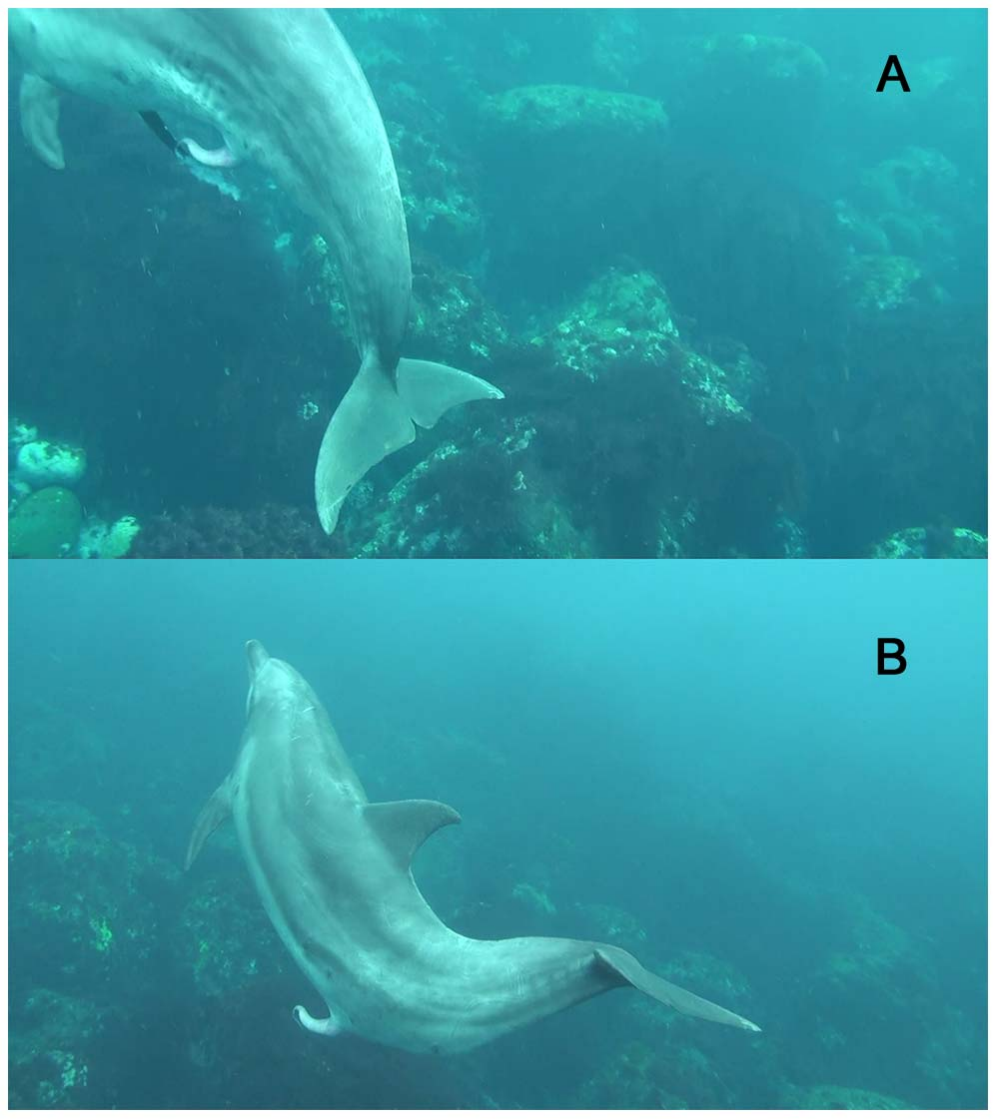
Spontaneous ejaculation by a wild Indo-Pacific bottlenose dolphin off Mikura Island, Japan. (A) Dense seminal fluid was ejaculated from the tip of the penis with initial contraction of the peduncle muscle downward. (B) A few seconds after the first ejaculation of seminal fluid, the remaining seminal fluid was ejaculated for 0.86 s (26 frames at (1/30 s)/frame).

Dolphin #266 was first filmed with another male (ID number #557; 11 years old), neither of which had an erect penis. At the time, neither #266 nor #557 was moving their peduncle flukes and both were swimming by inertia. The left eye of #557 was closed. Ten seconds later, #266 was filmed again with a fully erect penis without peduncle fluke movement, and with a closed left eye. The eye opened at 3.2 s (96 frames at (1/30 s)/frame) before spontaneous ejaculation. #266 seemed to move his peduncle flukes gently downward 1 s before spontaneous ejaculation. Spontaneous ejaculation lasted 0.43 s (13 frames at (1/30 s)/frame) with the contraction of the peduncle muscle downward and dense seminal fluid being ejaculated from the tip of the penis (Figure 1A and Video S1). The dolphin stretched his peduncle muscle upward and a few seconds later the remaining seminal fluid was ejaculated, lasting 0.73 s (22 frames at (1/30 s)/frame) (Figure 1B and Video S1). After ejaculation, the dolphin gently swam away.

### Behavioral and group states

We counted 37 dolphins in this group (including #266) both from the boat and underwater during the 7-min observation. Twenty-nine out of the 37 dolphins were identified and consisted of 7 mother and calf pairs (including 3 neonates), 6 males, and 9 females without a calf. The group state was “resting”, as dolphins swam slowly and quietly in the same direction with few breaths, but with a relatively widely spread “carpet formation”. Sexual behavior, and sociosexual behavior were not observed. No male dolphin except #266 showed his penis. There were no audible “pop” sounds, which are directed by male dolphins toward females during consortship [35], heard during the observation period. Most females and calves were in the front, with several males following, and #266 and #557 were at the rear of the group.

## Discussion

This is the first report of spontaneous ejaculation in an aquatic mammal. Spontaneous ejaculation has previously been reported in boreoeutherian mammals. As Beach [10] suggested, spontaneous ejaculation, including in human males, may have an ubiquitous physiological function considering the phylogenetically widespread nature of this phenomenon.

Spontaneous ejaculation in dolphins seems to be a very rare event, similar to other mammals except Rodentia; none of the researchers involved in dolphin research in Mikura Island had previously observed this behavior during our vast number of observational experiences, either in the wild or in various aquariums, and there are no published reports of this phenomenon. No spontaneous ejaculation was found in the previous 13,062 min of video data collected from 1994 to 2012 for the ID studies at Mikura Island.

Group behavioral state was resting, and we observed the closed left eye of #266, an indication of unihemispheric (or bilateral) sleep (reviewed in [36]), just before spontaneous ejaculation. On the basis of these observations, we assume that #266 was in a “drowsy state” when he spontaneously ejaculated. The spontaneous ejaculation in dolphins reported here thus could have “no clear function or misuse of inhibitory neural control system during drowsiness and sleep,” which is categorized as (3) in Introduction. Spontaneous ejaculations in various animals, including rats, guinea pigs, domestic cats, warthogs, horses, chimpanzees, and humans occur when drowsy or asleep [3], [5], [7]–[9], [11], [18], [37]. Although Kinsey et al. [38] explained nocturnal emission in human males as “psychic stimulation during sleep,” some equally ubiquitous physiological function should exist given the phylogenetically widespread nature of nocturnal emission or spontaneous ejaculation [10]. During such states, the central nervous system (especially the inhibitory control region for the ejaculation) may be partially relaxed [11], [17], [18], [20].

No dolphin, including #266, showed any masturbation activity such as genital rubbing on objects which has been reported in various dolphin species [26]–[31]. Furthermore, no sexual or socio-sexual behavior was observed, and no dolphin showed his penis during the observation period in spite of it being the high breeding season. Therefore, the spontaneous ejaculation reported here did not directly relate to masturbation or sexual or socio-sexual activity. As no females were within eyesight around #266, and #577 was in front of #266 when the spontaneous ejaculation occurred, the spontaneous ejaculation reported here also could not have functioned as a sexual display.

No research has been conducted to reveal the mechanism of ejaculation in dolphins. If the mechanism is similar to other animals such as rats, the neural control of ejaculation in dolphins might be located at the spinal level and controlled by the androgen- and gastrin-releasing peptide system [39]–[41]. During drowsiness, this neural control system would be partially relaxed, and spontaneous ejaculation could occasionally occur in dolphins.

Although we did not verify that the fluid we observed was actually an ejaculate, there is little possibility that the fluid was other substance such as urine or purulent matter when taking into account the white color, thickness, and pulsive emission of the fluid with a fully erect penis.

Reports of spontaneous ejaculation from various animals are needed to understand this phenomenon, including in human males, and to reveal its evolutionary function. It is difficult to observe spontaneous ejaculation, which only lasts a few seconds in animals; however, collecting such information is important for an understanding the animal basis of spontaneous ejaculation, or “wet dreams” in human males.

## Supporting Information

Video S1 **Spontaneous ejaculation by a wild Indo-Pacific bottlenose dolphin (** ***Tursiops aduncus*** **) off Mikura Island.**
(MP4)
